# Surface Plasmon Resonance Biosensor Based on Smart Phone Platforms

**DOI:** 10.1038/srep12864

**Published:** 2015-08-10

**Authors:** Yun Liu, Qiang Liu, Shimeng Chen, Fang Cheng, Hanqi Wang, Wei Peng

**Affiliations:** 1 School of Physics and Optoelectronic Engineering, Dalian University of Technology, Dalian 116024, China; 2 State Key Laboratory of Fine Chemical; 3 School of Pharmaceutical Science and Technology, Dalian University of Technology, Dalian 116024, China

## Abstract

We demonstrate a fiber optic surface plasmon resonance (SPR) biosensor based on smart phone platforms. The light-weight optical components and sensing element are connected by optical fibers on a phone case. This SPR adaptor can be conveniently installed or removed from smart phones. The measurement, control and reference channels are illuminated by the light entering the lead-in fibers from the phone’s LED flash, while the light from the end faces of the lead-out fibers is detected by the phone’s camera. The SPR-sensing element is fabricated by a light-guiding silica capillary that is stripped off its cladding and coated with 50-nm gold film. Utilizing a smart application to extract the light intensity information from the camera images, the light intensities of each channel are recorded every 0.5 s with refractive index (RI) changes. The performance of the smart phone-based SPR platform for accurate and repeatable measurements was evaluated by detecting different concentrations of antibody binding to a functionalized sensing element, and the experiment results were validated through contrast experiments with a commercial SPR instrument. This cost-effective and portable SPR biosensor based on smart phones has many applications, such as medicine, health and environmental monitoring.

In recent years, there has been increased demand for the development of simple, light-weight, low-cost and rapid detection devices for applications related to clinical diagnosis^1^, health care^2^ and environmental monitoring^3^, not only for scientific research but also for daily work^4^,^5^,^6^,^7^. The emergence of portable mobile devices with internet connectivity, high-resolution cameras, touch-screen displays, and high-performance CPUs has facilitated the development of devices suited for out-of-the-laboratory applications without dedicated instruments and laboratory conditions for sensing, detection and analysis. The availability of portable mobile devices with internet connections led to the design of chemical sensors^8^,^9^. Because smart phones are ubiquitous, integrating biosensing devices into smart phones is a promising approach for the creation of a pervasive biosensor for public health and environmental protection. Recently, many smart phone-based sensing platforms have been developed that provide a simple method for point-of-care testing (POCT), clinical diagnosis of diseases, and detection of pathogens^10^,^11^,^12^. Thus, smart phone hardware and software lend themselves the development of sensing platforms for optical biosensors.

Some analytical methods intended for smart phones demand light sources with high intensities or monochromatic lights that cannot be achieved by a phone flash. Hence, the illumination of smart phone-based sensors cannot always rely on the phone flash. In these cases, additional battery and light sources are necessary, such as light-emitting diodes (LED) or semiconductor lasers^13^,^14^, leading to inefficient utilization of smart phones. Optical alignment is critically important for optical biosensing because it directly affects the accuracy, repeatability and reliability of measurements. Moreover, handling of the sensor during chemical treatments, washing, and contact with reagents may cause misalignment. Because these sensors are intended for untrained personnel, these issues must be addressed.

Herein we demonstrate a portable fiber-optic surface plasmon resonance (SPR) biosensor using a smart phone as a sensing platform. This SPR sensor employs surface electromagnetic evanescent waves at the metal dielectric interface and is highly sensitive to changes in the small refractive index (RI)^15^. Over the past decade, many SPR sensors have been reported in applications such as biomolecular interaction analysis, medical diagnostics, environmental monitoring, and food safety^16^,^17^,^18^. Traditional SPR devices generally require expensive equipment, complicated optics, and precise alignment of the components^19^,^20^, features that hinder the development of a portable device. Current portable SPR devices still require a portable computer to run the instrument and are about the size of a lunch box^21^. As an alternative to conventional bulk SPR sensors, fiber-optic SPR sensors have been proposed for the development of miniaturized SPR sensors^22^. Fiber-optic SPR sensors have distinct advantages for remote sensing, *in situ* measurement and *in vivo* measurement, including simpler structure, low cost, miniaturization, and smaller sample volume^23^,^24^. Various fiber-optic SPR sensors for chemical and biochemical sensing have been reported. Typical examples include optical-fiber SPR biosensors employing fibers such as multimode fibers (MMF)^25^, single-mode fibers (SMF)^26^, photonic crystal fibers^27^ and D-shaped optical fibers^28^ manufactured by side-polishing^29^, tapering, and changing the structure of the fiber end^30^, inscribing grating^31^, and other methods.

In this work, we used the fiber-optic SPR (FO-SPR) structure, which greatly enhances the simplicity and flexibility of the optical alignment and light coupling compared with a prism-based SPR configuration^32^,^33^, and demonstrated biosensing for a broad range of analytes^34^,^35^,^36^. FO-SPR does not require separate optical components such as prisms, slides, optical benches, or mechanical parts, which are large, expensive and limit system miniaturization, integration and portability. All optical components and the sensing element are connected by optical fibers and fixed on a phone case, which is designed to have a simple, light-weight, small structure adapted to smart phones and simple installation or removal. The measurement channel (MC), control channel (CC) and reference channel (RC) are illuminated by the light entering into the lead-in fibers from the phone LED flash, whereas the light from the end faces of the lead-out fibers is detected by the phone camera. The sensing elements are silica capillaries that have been stripped of their cladding and coated with 50-nm gold film. When the sample is injected into the flow cell, the light interacting with the sensing region is absorbed because of the SPR resonance, and the phone camera measures a corresponding intensity change of the light coming out of the lead-out fiber. A smart application extracts the light intensity information from the camera images at a frequency of 2 Hz for the MC, CC and RC. Because the sensing elements are packaged into a flow cell and the light is coupled by optical fiber connectors, optical path alignment, disassembly and assembly are easily and conveniently achieved. The sensing elements are sealed to prevent exposure and operator contact, and only the reagents or samples are pumped into the flow cell in sequence, either in the testing or the cleaning process. Thus, the operation was simplified to ensure reliable operation.

## Results

**Figure 1:**
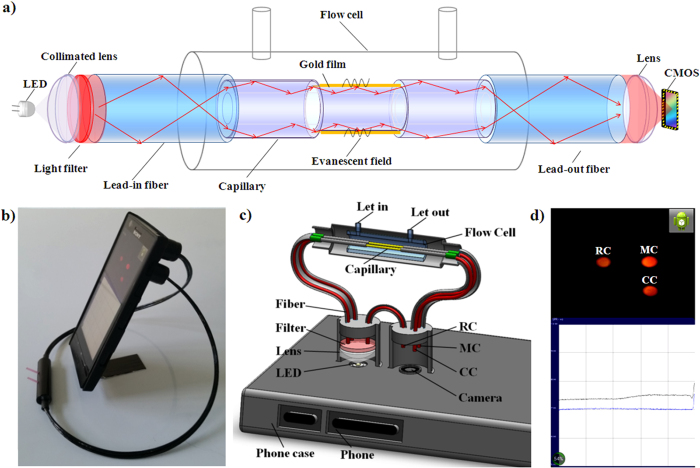
Instrumentation of the smart phone-based surface plasmon resonance imaging biosensor. (**a**) Schematic of the smart phone-based SPR sensor. (**b**) Photograph of the SPR sensor installed on an Android-based smart phone. (**c**) 3D schematic illustration of the internal structure of the opto-mechanical attachment. (**d**) The camera of the smart phone captures the images of the measurement channel, control channel and reference channel; then, the images are rapidly processed to obtain the relative intensity. The data points are plotted and displayed on the screen.

A schematic diagram, a photograph, and the running interface of the detection system are presented in Fig. 1(a–d). Because all components are fixed to the phone case (installed on the backside of the smart phone), the touch-screen interface and display are not affected during the detection process. In this case, the sensing components and the smart phone can be assembled together into an instrument and disassembled easily after the measurement. Before the lead-in and lead-out fibers (hard plastic cladding silica optical fiber, HPOF, HP 400/430-37/730E YOFC) are fixed, all end faces of the fibers are polished using emery paper. By fixing the fibers in the corresponding slot of the phone case, the camera and LED flash in the back of the phone can be aligned with the end faces of the lead-in and lead-out fibers. After the filter, a low-cost plastic lens is used to collimate the red light because the LED flash is a cold light source. To restrain the stray light, the lead-in and lead-out fibers are covered by a black rubber tube. The other ends of the lead-in and lead-out fibers are packaged into optical fiber connectors, which facilitate the connection of the lead-in and lead-out fibers with the sensing elements inside the flow cell and easy assembly and disassembly of the sensing elements. Because the light source in our SPR system is an LED flash light rather than the high-performance sources used in other SPR devices, the power instability of the LED flash was considered. Because this SPR sensor is designed for biosensing, one of the sensing elements is used as a measurement channel that has been functionalized, and another sensing element without functionalization is used as a control channel. To compensate for fluctuations in the intensity of the LED flash, we added another HPOF as a reference channel to monitor the light intensity of the LED flash. To ensure that the light entering the lead-in fibers and reference fiber are in same condition, the end of the reference fiber is fixed next to the end of the lead-in fibers; thus, any fluctuations of the LED flash affect both the measuring channel, control channel and the reference channel in the same manner simultaneously.

The smart phone SPR system uses intensity modulation. A narrow-band filter (center wavelength of 590 nm and full width at half-maximum (FWHM) of 8 nm) placed between the flash of the cell phone and the lead-in fibers provides nearly monochromatic incident light. Light then interacts with the SPR-sensing region and is analyzed by the camera of the cell phone. By monitoring the variation of the intensity of the light passing through the sensing elements, binding processes on the SPR sensor can be recorded, similar to imaging SPR. Hence, the smart phone SPR system is suitable for label-free and real-time optical detection of biological interactions and the estimation of the kinetic parameters of molecular interactions. An Android software application (App) was developed to simultaneously set the camera exposure, obtain images, and activate the LED flash light as the light source. The end faces of the measuring channel, control channel and reference channel captured by the camera are displayed as three light spots, which allows their light intensities to be calculated separately. Because fluctuations of the light intensity of the LED flash light affect the measuring channel, control channel and reference channel similarly, we used the relative intensity to effectively eliminate errors from the power fluctuations of the LED. Operating the App for measurement is easy because it is designed for touchscreen use. The operator needs only to touch the setting buttons at the top of the screen to control start/stop and pause/continue operations, adjusting the coordinates by left-right sliding and up-down sliding a finger across the touch screen to scale the curved profile, which is composed of a series of discrete data points for easy viewing in real time. The data for each test are stored by the App in text form in the phone memory card and can be retrieved and displayed. With the mobile web, the data also can be uploaded and exported to be shared, consulted and further analyzed.

**Figure 2:**
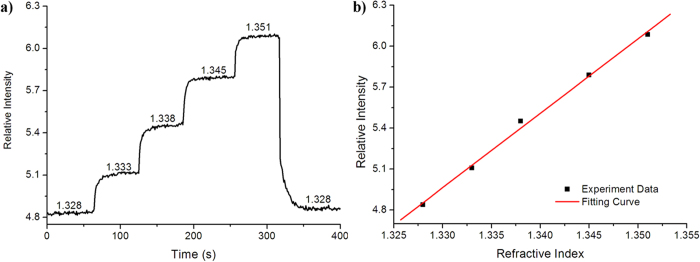
RI testing results for the prototype smart phone-based SPR imaging sensor. (**a**) Time response of the SPR sensor for solutions with different RIs. (**b**) Steady-state SPR response vs. refractive index units (RIU), from which the sensitivity and resolution were calculated.

Using a mini pump, the sensitivity and resolution of the SPR device were characterized by sodium chloride solutions with RIs of 1.328, 1.333, 1.338, 1.345, and 1.351 delivered into the flow cell at a constant flow rate of 180 μL/min (Fig. 2). The RIs of the sodium chloride solutions (calibration samples) were calibrated using an Abbe refractometer (WAY-2S). After fitting, the relative intensity had a linear response to the RI in the RI range of 1.328–1.351 (Fig. 2(b)), consistent with the range for most common biofluids. In the RI range of 1.328–1.363, the system provided a sensitivity of 1136%/RIU and a resolution greater than 7.4 × 10^−5^ RIU; the noise level is 0.08% and signal-noise ratio of the camera are better than 48 dB.

**Figure 3:**
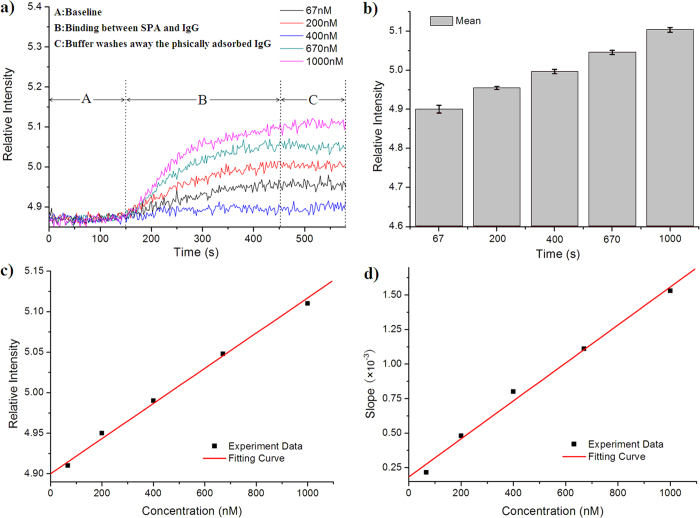
Biological interaction analyses using a capillary SPR sensing element integrated with a smart phone. (**a**) Interaction analysis of a capillary SPR sensing element functionalized and running on a smart phone for detection of IgG at concentrations of 67 nM, 133 nM, 200 nM, 400 nM, 670 nM and 1 μM. (**b**) Five different samples were assayed in repeated experiments. Each measurement at a given concentration was repeated three times. The mean and standard deviation were calculated. (**c**) The relationship between the measured relative intensity and sample concentration. (**d**) The relationship between the slope of the interaction curves and sample concentration.

**Figure 4:**
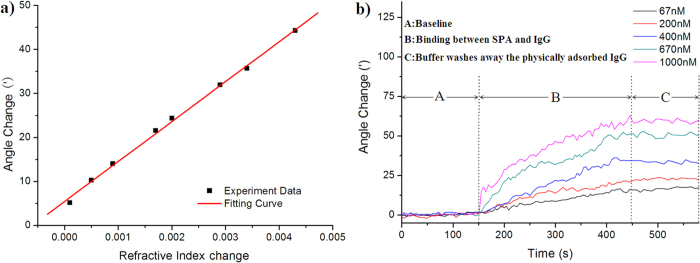
Biological interaction analyses using a commercial SPR instrument (Biosuplar 6). (**a**) Resolution was calculated from the steady-state SPR response vs. refractive index units (RIU). (**b**) Interaction analysis of the commercial SPR instrument for IgG detection at IgG concentrations of 67 nM, 133 nM, 200 nM, 400 nM, 670 nM and 1 μM.

To determine whether our smart phone SPR sensor can rely on the interrogation of relative intensity changes for analyte-sensing applications in real-time and dynamic monitoring, we performed experiments to examine the specific binding of Staphylococcal Protein A (SPA) and bovine immunoglobulin G (IgG) in solution on the sensing surface. Due to the interactions between SPA and Fc fragments of IgG, SPA is widely used as an immunological tool for antibody testing, purification and disease diagnosis. Figure 3 presents the analyte detection results. In this example, a capillary SPR sensor was functionalized with immobilized SPA to capture IgG. By regenerating the biosensor surface, various concentrations of IgG protein in buffer solution were evaluated. Figure 3(a) presents the variations in SPR relative intensity during the test procedure. As the binding-response curves indicate, the baseline (stage A) of our smart phone SPR sensor was very stable during the monitoring. The relative changes in the intensity of the SPR sensor’s responses to binding (stage B-C) were proportional in size to the increase in the concentration of IgG injected between 67 nM and 1 μM IgG, indicating that more IgG bound to SPA in the same period of time. The binding rate and amount of bound IgG were calculated from the slopes and relative intensity changes in the binding curves. The slopes (change in relative intensity per second) of the interaction profiles were evaluated at 150 s (the beginning of stage B in Fig. 3(a)), and the average relative intensity values were calculated from the values between 450 s and 575 s (stage C in Fig. 3(a)) of the interaction profile. By plotting the relative intensities and slopes in the diagrams shown in Fig. 3(b,c), we determined that the responses were approximately linear functions of the IgG concentration described by the linear relationship [Relative Intensity] = 4.901 + 2.469 × 10^−4^ [Concentration] and [Slope] = 1.855 × 10^−4^ + 1.373 × 10^−6^ [Concentration]. Therefore, by monitoring the relative intensity and slopes in an experiment, we can determine the concentration of IgG and obtain binding-kinetics information. This demonstrates that although the smart phone-based SPR sensor has a compact size and is easy to manipulate, it can be used as a portable detection device to obtain abundant information about analytes from micro-samples with a low concentration (in the nM range). These experiments also demonstrate a limit of detection of the smart phone SPR sensor of 47.4 nM, which we determined from the noise level and the relationship between the relative intensity response and IgG concentration. Furthermore, the detection limits of the smart phone SPR sensor can be increased using secondary antibodies^37^ or nanoparticles^38^ to enhance the SPR effect. To validate reliability and repeatability, each concentration of IgG was assayed three times using this SPR system, and the statistical results are summarized in Fig. 4(b). The standard deviations were less than 0.011. Thus, these experiments and subsequent statistical analysis verified the reliability of the detection system under laboratory conditions.

In addition, as a comparison between our smart phone SPR sensor and conventional SPR machines, the above RI measurements and biological detection of IgG were determined using a commercial SPR instrument (Biosuplar 6, Analytical *μ-*Systems, Germany). The RI response of the commercial SPR instrument is presented in Fig. 4(a) and yielded a resolution of 2.7 × 10^−5^ RIU. The binding response curves are presented in Fig. 4(b). The experimental results demonstrated that the commercial SPR instrument’s SPR resonance-angle responses to the binding process (stage B) were also proportionally larger with the injection of increasing IgG concentrations from 67 nM to 1 μM, similar to the binding-response curves obtained with the smart phone SPR sensor. The average angle-change values were used as the measured value to characterize the amount of bound IgG from 450 s to 575 s (stage C) of the interaction profile. An approximately linear function was observed between the angle-change response and IgG concentration, described by [Angle change] = 12.621 + 4.685 × 10^−2^ [Concentration]. Using the noise level and the relationship between the angle change and IgG concentration, we determined that the detection limit of the commercial SPR instrument was 15.7 nM. The key performance indicators of the commercial SPR instrument and the smart phone SPR sensor are presented in Table 1. This analysis indicated that the RI resolution and IgG detection limit of the commercial SPR instrument were superior to those of the smart phone SPR sensor but the same order of magnitude. However, our smart phone SPR sensor has the apparent advantages of being low-cost, compact, and lightweight with facilitation and generalization, important improvements of the SPR biosensor.

## Discussion

### Principle of smart phone-based SPR sensors

**Figure 5:**
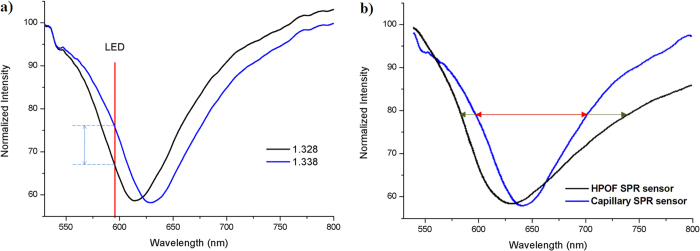
Transmission spectrum of the capillary SPR sensor. (**a**) Spectral response of the capillary SPR sensor for solutions with RIs of 1.328 and 1.338. (**b**) FWHM of the capillary and HPOF SPR sensors.

The operating principle of the smart phone SPR system relies on the penetration of the filter by the light from the smart phone flash and transmission to the lead-in fibers (Fig. 5). The 5-mm coating and cladding of the capillary was removed, and a gold film of 50 nm was coated onto the sensing region. Hence, when light reaches the sensing region of the capillary, some propagation modes enter resonance with the gold film due to the SPR effect. Because the SPR absorption depends on the dielectric properties of the thin layer of solution near the surface of the sensor region, we can monitor and analyze the binding interactions of the sample with SPR sensing. Thus, after calibration, the SPR device can measure samples quantitatively.

The absorption spectra of the SPR sensor were measured and recorded in solutions with RIs of 1.328 and 1.338 (Fig. 5(a)). As expected, the absorption spectrum shifted to longer wavelengths for higher RIs. By recording SPR at a single wavelength (590 nm) and angle couple (dictated by the propagation modes of the FO), the power output changed with changes in RI. The selection of material for the SPR sensing element is important; HPOF was investigated for use in the lead-in and lead-out fibers and capillaries, but comparison of transmission spectra and the wavelength response to RI changes revealed that the capillary SPR sensor had a narrower FWHM (Fig. 5(b)) but similar wavelength sensitivity compared with the HPOF SPR sensor. Consequently, a steeper falling edge can be used for intensity modulation with higher intensity sensitivity. The main reason a capillary SPR sensor was selected over an HPOF SPR sensor is that the capillary has a smaller NA than the HPOF and supports fewer modes. Thus, light reaches the sensing region with fewer incidence angles, leading to a narrower FWHM.

### Digital processing of images by the Android application

An Android software application (App) was developed to specify the camera exposure to allow image collection and the LED flash light as the light source at the same time. Because the end faces of the measuring channel, control channel and reference channel captured by the camera were displayed as three light spots, their light intensities were calculated and compared separately. Because the light intensity fluctuations of the LED flash light affect the measuring channel, control channel and reference channel similarly, we used the relative intensity to effectively eliminate errors from the power fluctuations of the LED. The relative intensity was expressed as I_R_ = (I_m_ − I_c_)/I_r_, where I_m_, I_c_ and I_r_ are the intensity values of the measuring channel, control channel and reference channel, respectively. Because we provide a simple and feasible compensation method using a capillary SPR sensor without chemical modification and an HPOF as the control channel and reference channel and calculating the relative intensity as the output signal, the influence of the bulk refractive index and power instability can be excluded. To extract the brightness information from the image data by the App, the colored image was converted into a gray-scale image. Every image point has a gray-scale value between 0% (white) and 100% (black) to indicate its brightness. Therefore, the light intensity of a photograph can be calculated by integrating the gray-scale value of the measurement and reference spots. To conveniently check the light spots of the measuring channel, control channel and reference channel, the image monitored by the phone camera is displayed as thumbnails in the touch screen interface. Under the thumbnails, the light intensity data are captured and processed every 0.5 seconds and presented as intensity-time coordinates in real time.

In this study, a portable SPR detection instrument based on a smart phone and its biomolecular detection capabilities were demonstrated. The SPR detection platform employs fiber-optic SPR as a sensing element and a smart phone as the light source and detector. The sensing element and optical couplers were based on optical fiber components, which have high sensitivity and good portability, leading to an SPR instrument that can perform precise detection without complex, dedicated, specialized and fragile light elements or sophisticated optical calibration. A reference channel was added to eliminate the effect of LED instability of the flash and increase the accuracy and reliability of the instrument. All optical components were inexpensive and fixed on the phone case for convenient installation on the smart phone. The optical setup can be adapted to different platforms, such as Android phones and iPhones. The sensitivity and repeatability of the SPR platform were evaluated by detecting different concentrations of antibody binding to a functionalized sensing element. The advantages of the smart phone SPR sensor includes simple, rapid, efficient, sensitive, and accurate operation, good repeatability, and regeneration. To demonstrate the performance of the smart phone-based SPR biosensor, a layer of Protein A was immobilized on the surface of the gold film, and the SPR biosensor was used for the specific detection and evaluation of the bovine IgG protein concentration, which has important applications in disease diagnosis and biological drug manufacturing. Comparison with commercial SPR instruments not only validated our experimental results and sensitivity but also demonstrated that our smart phone SPR sensor provides an effective and efficient biosensing method. Because the sensing element can be modified for diverse SPR experiments and smart phones can be connected to the Web, a cost-effective and portable SPR biosensor based on smart phones will be useful for point-of-care tests, ubiquitous healthcare, and environmental monitoring and has potential as a tool for analyzing molecular interactions.

## Methods

### Smart phone-based SPR sensor fabrication

The proposed SPR sensor benefits from light-guiding flexible capillary (LTSP 150/375 Polymicro) SPR sensors as the sensing elements and light coupling with HPOF. The inner diameter and outside diameter of the capillary were 150 μm and 375 μm, respectively, and its numerical aperture (NA) was 0.22. The core and cladding diameters of the optical fiber were 400 μm and 430 μm, respectively, and its NA was 0.37. The smart phone used as a sensor platform in this experiment was an Android phone (SONY Lt22i, Dual Core ARM Mali-400 MP Processor, 8 megapixel camera). A commercial phone case was used as a holder to carry all of the optical elements, including the filter, collimation lens, lead-in fibers, and lead-out fibers. Two plastic pipes facing the camera and the LED flash were glued to the phone case, which were used to contain the filter and collimation lens and fix the fibers. To eliminate the influence of environmental light, the outside surface of the plastic pipes was painted black.

### Reagents

11-Mercaptoundecanic acid (11-MUA), N-hydroxysuccinimide (NHS), 1-ethyl-3-(3-dimeth-ylamino-propyl) carbodiimide (EDC), and protein A from *Staphylococcus aureus* were obtained from Sigma. Bovine immunoglobulin G (IgG) was obtained from Merck KgaA (Darmstadt, Germany). Bovine serum albumin (BSA) was obtained from Melonepharma (Dalian, China). Other chemicals were of analytical grade from local sources.

### Functionalization

The capillary SPR sensors (Smart phone-based SPR sensor) and SPR chip (Biosuplar 6) were functionalized with an amine-coupling reaction to facilitate the attachment of Protein A. They were soaked in 1 mM 11-mercaptoundecanic acid (MUA) in ethanol at room temperature for 12 hours to form a carboxyl surface and then treated with a solution of 0.5 M N-hydroxysuccinimide (NHS) and 0.55 M 1-ethyl-3-(3-dimethylamino-propyl) carbodiimide hydrochloride (EDC) in ultrapure water at 4 °C for 30 minutes to convert the carboxyl surface to activated ester. The treated SPR sensors were dipped in Protein A (0.1 mg/ml in 0.01 M phosphate-buffered saline (PBS; pH = 7.4)) for 30 minutes. Unreacted ester groups were blocked by bovine serum albumin (BSA, 1 mg/ml in PBS) at room temperature for 15 minutes.

### Test procedures

After Protein A immobilization and BSA blocking, the sensing elements were assembled with a flow cell and aligned with lead-in fiber and lead-out fibers for measurement. Because the SPR sensor was designed as a portable tool, all operations should be easy and feasible for field applications. To investigate the response of the sensor to IgG, different concentrations of IgG of 67 nm to 1 μM in PBS were separately pumped into the flow cell at a flow rate of 180 μl/min using a mini-pump (Longer, T60-S2&WX10-14, dimensions: 107 × 60 × 80 mm, weight: 450 g). For each assay, the volume of IgG sample is 1 mL. The relative intensity increased significantly due to IgG binding specifically to Protein A on the sensing region surface, and the values of the relative intensity were recorded and plotted as a function of time on the phone screen (Fig. 3a). Then, PBS was pumped into the flow cell to remove unbound IgG molecules, which resulted in a slight reduction in the relative intensity. Urea solution (8.0 M) was then used to strip the surface-bound IgG and effectively regenerate the sensing region after the sensing element was rinsed with PBS. The same procedure was applied to the commercial SPR instrument (Biosuplar 6).

## Additional Information

**How to cite this article**: Liu, Y. *et al.* Surface Plasmon Resonance Biosensor Based on Smart Phone Platforms. *Sci. Rep.* **5**, 12864; doi: 10.1038/srep12864 (2015).

## Figures and Tables

**Table 1:** Comparison of commercial SPR instrument and proposed SPR biosensor results for RI measurements and biological IgG detection.

	Commercial SPR instrument(Biosuplar 6)	Smart phone SPR biosensor
**Resolution**	2.7 × 10^−5^ RIU	7.4 × 10^−5^ RIU
**Limit of detection**	15.7 nM	47.4 nM
**Cost**	220,000¥	240¥
**Size (without computer/smart phone)**	20 cm × 9 cm × 8 cm	12 cm × 6 cm × 2 cm
**Weight (without computer/smart phone)**	2.5 kg	40 g
**Computer/smart phone and operating system requirements**	PC, Windows 98/2000 or Windows XP	Smart phone, Android
